# Supplementary material for: Essential Involvement of Neutrophil Elastase in Acute Acetaminophen Hepatotoxicity Using BALB/c Mice
Source: Int J Mol Sci. 2023 Apr 25;24(9):7845. doi: 10.3390/ijms24097845 (PMC10177873; doi:10.3390/ijms24097845)
Supplement: Supplementary file 1 [file ijms-24-07845-s001.zip › ijms-2184666-supplementary.pdf]

Methods

In vitro culture and stimulation of RAW264.7

RAW264.7, a mouse macrophage cell line, was seeded on a 24-well plate at 1×10^5 cells/well and incubated at 37°C in 5% CO_2 in DMEM supplemented with 1% FBS and 1% penicillin/streptomycin for 3 d to confluence. Prior to NE treatment, the cells were washed twice with 1 ml of PBS and cultured for 1 h with the corresponding serum-free medium. RAW264.7 cells were treated with 0.5 μg/ml NE for 16 h, washed twice with 1 ml of PBS, and cultured at 37°C in 5% CO_2 in fresh serum-free medium for 6 h before harvesting.

Results

Gene expression of inflammatory cytokines and CXC chemokines in NE-treated macrophages

Macrophages are main producers of inflammatory cytokines and chemokines during APAP-induced liver injury. To understand the role of NE in inducing inflammatory cytokine and CXC chemokine expression, mice RAW264.7 macrophages were exposed to purified NE, and gene the expressions for IFN-γ, TNF-α, IL-1β, CXCL1, and CXCL2 were evaluated by real-time RT-PCR. The expression of all examined gene was found to be significantly increased in the NE-treated macrophages in vitro (Supplemental figure 1). These results support in vivo observations and indicate that NE can mediate the induction of expression of IFN-γ, TNF-α, IL-1β, CXCL1, and CXCL2 in macrophages.

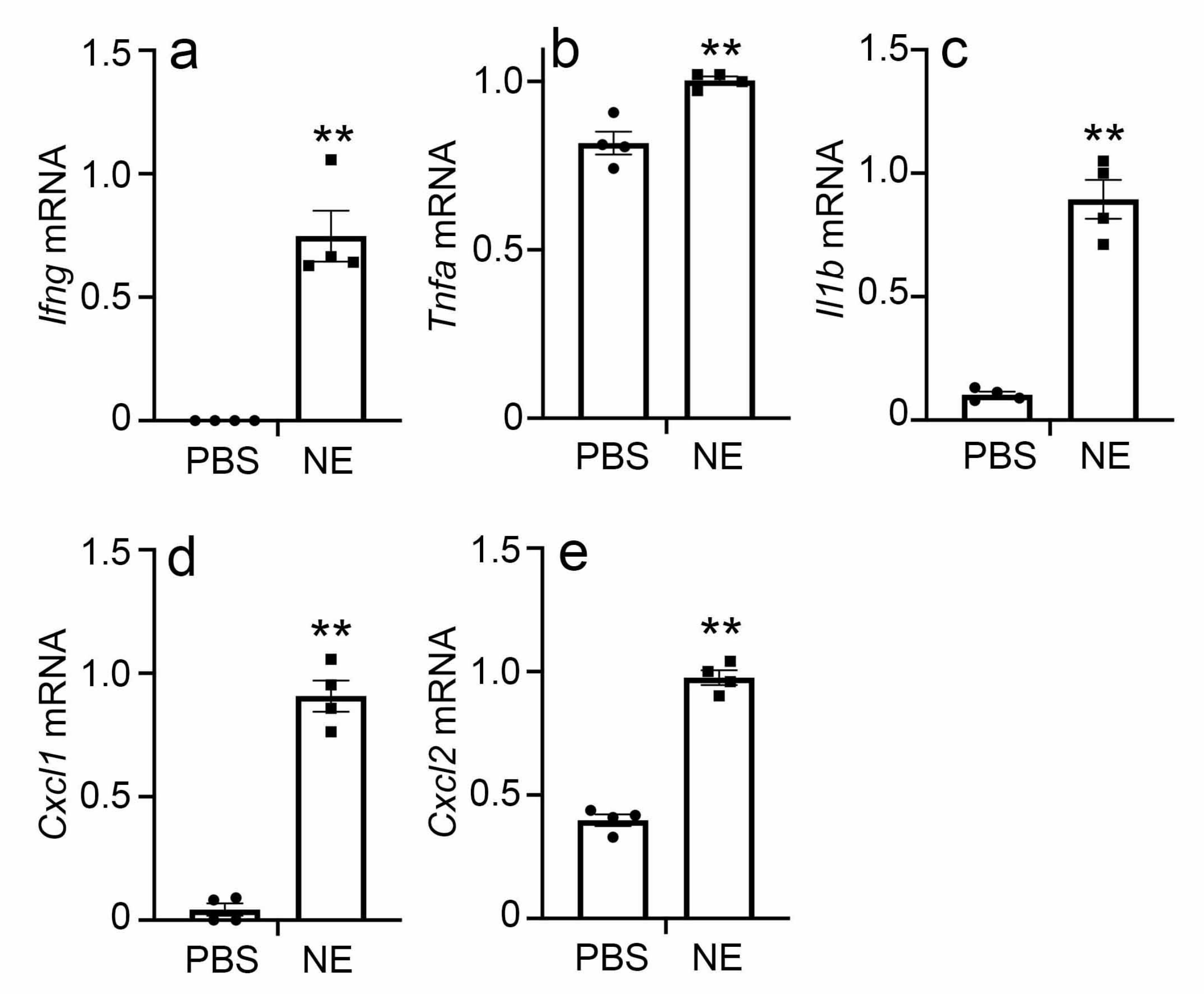

Supplementary Figure S1. Effect of NE on expression of inflammatory cytokines and CXC chemokines in macrophages. *Ifng* (a), *Tnfa* (b), *Il1b* (c), *Cxcl1* (d), and *Cxcl2* (e). All values represent means ± SEM (four independent experiments). *P < 0.05; **P < 0.01, vs. PBS treatment.
